# Clinical outcome and physical activity measured with StepWatch 3™ Activity Monitor after minimally invasive total hip arthroplasty

**DOI:** 10.1186/s13018-018-0775-4

**Published:** 2018-06-15

**Authors:** Steffen Höll, Andreas Blum, Georg Gosheger, Ralf Dieckmann, Corinna Winter, Dieter Rosenbaum

**Affiliations:** 1 Movement Analysis Laboratory, Institute of Experimental Musculoskeletal Medicine IEMM, University Hospital Münster, Domagkstrasse 3, 48149 Münster, Germany; 2 Department of Orthopedics and Tumor Orthopedics, University Hospital Muenster, Albert-Schweitzer-Str. 33, 48149 Münster, Germany

**Keywords:** Minimal invasive total hip arthroplasty, Physical activity, Gait cycles, StepWatch 3™ Activity Monitor

## Abstract

**Background:**

Subjective data and physical examinations of patients after total hip arthroplasty are used to assess the outcome. But regarding the physical activity, no objective data can be delivered by existing scores. The level of activity can be measured objectively by counting gait cycles. The aim of this study was to measure activity levels of patients before and after total hip arthroplasty (THA).

**Methods:**

Forty-six patients were included in this prospective study. Western Ontario and McMaster Universities Arthritis Index (WOMAC), Harris Hip Score (HHS), and physical activity level based on the number of steps per day were assessed 1 week before surgery, 6 weeks postoperatively, and 3 months postoperatively. To assess the general constitution of the patients, the American Society of Anesthesiologists (ASA) score and BMI were determined. The physical activity level was measured by StepWatch 3™ Activity Monitor (SAM; Orthocare Innovations, Seattle, WA, USA). The number of GCs per day was assessed. Spearman’s rank correlation coefficients were used to identify an association between age, body mass index (BMI), and American Society of Anesthesiologists classification with the number of gait cycles and to detect correlation between GCs and HHS and GCs and WOMAC.

**Results:**

From preoperatively to 6 weeks postoperatively, the number of gait cycles did not alter significantly. Three months postoperatively, the number of GC/d and GC/h improved significantly. HHS and WOMAC improved significantly from before surgery to 6 weeks and to 3 months follow-up. The number of gait cycles per day did not correlate with the HHS and the WOMAC score at any point of measurement. Age, BMI, and ASA classification did not influence the results.

**Conclusion:**

By using a StepWatch 3™ Activity Monitor objective data about physical activity before and after THA can be measured reliable. Subjective and objective data in the postoperative period show different results. Physical activity seems to take longer to reach significantly improved values. By counting gait cycles, surgeons do have an additional tool to measure success after THA.

## Background

For patients with osteoarthritis of the hip, total hip arthroplasty (THA) is a highly successful procedure in end stage of the disease. Pain relief, functional recovery, and improving quality of life are the most important factors for the patients. Subjective data of the patients and physical examinations are used to assess the outcome after THA. There are many studies in which it could be shown that after hip arthroplasty, the patients improved significantly measured by scores like Western Ontario and McMaster Universities Arthritis Index (WOMAC) and Harris Hip Score (HHS) in a short and long term [1, 2]. Several studies could show good results after minimal invasive THA in the early postoperative period measured by HHS and WOMAC [3–5].

But regarding the physical activity, no objective data can be delivered by existing scores to show improvements after THA. In the literature, the level of activity can be measured objectively by counting gait cycles. Furthermore, recommendations on how many steps per day do represent an active life style can be done [6].

The aim of this study was to measure activity levels of patients with osteoarthritis of the hip before minimal invasive surgery (MIS) THA and after MIS THA. Cofactors influencing the results should be identified. Objective results were compared with existing scoring systems.

## Methods

Forty-six patients (female *n* = 25, male *n* = 21, mean age 63.3 ± 10.5 years, mean BMI 27.1 ± 4.0 kg/m^2^) with THA in minimal invasive technique using the direct anterior approach were included in this prospective study. The indications for THA were osteoarthritis *n* = 43 and femoral head necrosis *n* = 3. Postoperative protocol included full weight-bearing from the first postoperative day.

In 37 patients, cementless fixation was used and in 9 patients, hybrid fixation was used. In all patients, 36-mm ceramic heads with X3™ polyethylene, (Stryker Orthopedics) were used. Exclusion criteria were patients with femoral neck fractures, hip arthroplasty because of metastasis or tumor diseases, and patients with partial weight-bearing postoperatively.

WOMAC [1], HHS [2], and physical activity level (PAL) based on the number of steps per day were assessed 1 week before surgery, 6 weeks postoperatively, and 3 months postoperatively. To assess the general constitution of the patients, the ASA score and BMI were determined [7]. The PAL was measured by StepWatch 3™ Activity Monitor (SAM; Orthocare Innovations, Seattle, WA, USA). The patients worn the StepWatch 3™ for 1 week preoperative, 6 weeks postoperative, and 3 months postoperative. The device is an uniaxial accelerometer, which can be worn comfortably above the ankle due to its low size and weight (7.5 × 5 × 2 cm, 43 g). The SAM stores the number of gait cycles (GC, one GC = 2 steps) in intervals of 1 min. It gives no feedback and cannot be manipulated by the subject. The SAM is well validated and has an accuracy of 99% in detecting steps in intervals of 1 min [8, 9]. Other accelerometers have also shown that objective data can be collected to assess physical activity levels [10]. Patients were instructed to wear the SAM at the right leg lateral above the lateral malleolus for the whole day from getting up to going to bed and were asked to record the non-wear times. The SAM was programmed according to the individual height and gait dynamics of the patient. The number of GCs was assessed in intervals of 1 min and transferred to a computer via the StepWatch™ USB Dock. The number of GCs per day and the number of GCs per hour were assessed taking into account the different wear times. Movement intensity was defined by the number of gait cycles per minute.

Minutes with more than 50 GCs were counted as moderate-to-vigorous physical activity (MVPA) [6]. Wear times and duration are listed in Table 1.

**Table 1:** Wear times per week and per day, 1 week preoperatively, 6 weeks postoperatively, and 3 months postoperatively

	1 week preoperatively	6 weeks postoperatively	3 months postoperatively
Wear duration (days)	6.54 (± 0.75)	6.53 (± 0.88)	6.65 (± 0.62)
Wear time (h/d)	12:58 (± 1:28)	12:45 (± 1:08)	13:10 (± 1:18)

Trends in improvement of longitudinal data of activity parameters, WOMAC and HHS were analyzed using generalized estimating equations (GEE). Statistical significance was set at *p* value < 0.05. All statistical data analyses were created by IBM SPSS Statistics (version 19 or 20).

Spearman’s rank correlation coefficients were used to identify an association between age, body mass index (BMI), and American Society of Anesthesiologists (ASA) classification with the number of gait cycles (GCs) and to detect correlation between GCs and HHS and GCs and WOMAC.

## Results

From preoperatively to 6 weeks postoperatively, the number of gait cycles did not alter significantly. Three months postoperatively, the number of GC/d and GC/h improved significantly by 18 and 15% compared to the preoperative measurement (*p* < 0.05). The number of minutes with more than 50 GCs did not change at 6 weeks follow-up (*p* = 0.372), but at 3 months, it increased significantly by 58% (*p* < 0.01).

HHS improved significantly from 50 (± 13) before surgery to 82 (± 13) at 6 weeks and to 93 (± 6) at 3 months follow-up (both *p* < 0.01).

The WOMAC score increased significantly from 42 (± 19) before surgery to 76 (± 15) at 6 weeks (*p* < 0.01) and to 82 (± 13) at 3 months follow-up (*p* < 0.01) gait cycles, and scores are listed in Table 2.

**Table 2:** Gait cycles and scores preoperatively and at 6 weeks and 3 months follow-up

	GC/day	MVPA (minutes with more than 50 GC)	HHS	WOMAC
1 week preoperatively	4015 (± 1539)	3.1 (± 4.5)	50 (± 13)	42 (± 19)
6 weeks postoperatively	3724 (± 1543) *p* = 0.183	3.8 (± 5.1) *p* = 0.372	82 (± 13) *p* < 0.01	76 (± 15) *p* < 0.01
3 months postoperatively	4756 (± 1733) *p* < 0.05	6.0 (± 7.4) *p* < 0.01	93 (± 6), *p* < 0.01	82 (± 13) *p* < 0.01

Between patients younger than 65 and older than 65, no significant differences could be found in GCs per day at any point of measurements.

BMI did not influence number of GC (group 1 BMI < 30, group 2 BMI > 30).

The ASA classification did not influence the number of GC per day.

The number of gait cycles per day did not correlate with the HHS and the WOMAC score at any point of measurement.

## Discussion

Total hip arthroplasty is a very common and successful procedure in patients with end-stage osteoarthritis. For the patients’ reduced pain, improved physical function and activity, and regained quality of life are the most important factors after the procedure. By using scoring system pain, physical function and subjective physical activity are measured by the surgeons to evaluate the success of THA [1, 2, 11]. From other studies, we know that counting gait cycles per day is a possibility to get information about physical activity by objective measurements [6, 12].

Tudor-Locke stated that 10,000 steps/day are necessary for an “active” lifestyle. As expected, mobility with end-stage osteoarthritis is restricted. This restricted mobility is also reflected in significantly lower results in HHS and WOMAC. In terms of active, healthy lifestyle, the 4015 GCs found preoperatively in our study can be considered as “somewhat active” [6]. This activity level was comparable with that reported by Winter et al. for patients with end-stage osteoarthritis of the hip (3994 GCs) [13].

After THA, it is expected that pain is reduced. With reduced pain function, the joint is expected to get better. These things are the precondition for a more active behavior. Therefore, it is comprehensible that an increased mobility (gait cycles/day) can only be measured at a later onset than parameters like pain and function measured by the used scores.

In a study of end-stage hip and knee osteoarthritis patients, the authors came to the conclusion that the patients’ perception of physical functioning does not always correspond to the objective measured physical activity [14]. These results can be confirmed. In our study group, no correlation could be found between WOMAC, HHS, and gait cycles per day. In another study of total hip and knee arthroplasties, subjective parameters like pain relief, stiffness, and self-reported physical function showed a large improvement postoperatively. In contrast, the objective results of physical activity measured by an activity monitor with accelerometers were less than expected by 6 months after surgery respectively, and no improvement after 6 months could be measured [15–17].

Our patients could increase their objective physical activity significantly 3 months after surgery with 4756 GCs per day. After 6 weeks, GCs per day showed no significant improvement, whereas HHS and WOMAC showed significant improvements already after 6 weeks and again after 3 months.

Similar results at 8 weeks follow-up were reported by Brandes et al. for patients undergoing total knee replacement, and activity levels at this point of time were similar than preoperative whereas the knee society score improved significantly after 8 weeks [18].

In another study of patients after TKA, SF 36 and the Physical Activity Scale for the Elderly (PASE) increased significantly already after 3 months after the operation. The number of steps per day was 2693 at 3 months and 3518 at 6 months after the operation [19].

In 2016, Toogood et al. compared patients after minimal invasive (MIS) THA with conventional THA 30 days after the operation. In average, the number of steps per day was 2563 and in the MIS group, the level of activity was significantly higher [20].

In our collective, the activity level after 6 weeks and 3 months was higher than in the studies mentioned above. Although we could not found any differences in GCs/per day independence of age, patients in the other studies were about 10 years older in average [19, 20]. From the literature, we know that steps per day decrease with increasing age and physical activity decreases with age [12, 21].

But also, the MIS approach can be a reason for a higher activity level postoperatively.

Other factors like comorbidities and BMI with influence on activity levels are discussed in the literature. In our patients, ASA and BMI had no influence on objective activity level. Toogood et al. also showed that BMI greater or less than 30 kg/m^2^ had no influence on objective activity level [20]. In contrast to these results, self-reported physical activity at 1–2 years postoperatively after THA and TKA was lower depending on BMI, female sex, and specific comorbidities [22].

There are some limitations of our study. Although we used a prospective design, no control group was included. So, we cannot say if MIS surgery has a positive influence on activity level. The group of patients included was small. As a university hospital, most of the patients did not fulfill the inclusion criteria. Because we used the ASA classification, no information about comorbidities in detail were available. So, we could not detect individual diseases, which might have a negative influence on the level of activity.

The follow-up was limited to 3 months postoperatively, although we know that results do also improve in the following months [15].

## Conclusion

By using a StepWatch 3™ Activity Monitor, objective data about physical activity before and after THA can be measured reliable. As shown, subjective and objective data in the postoperative period show different results and although scores improve significantly already 6 weeks after the operations, objective physical activity seems to take longer to reach significantly improved values. By counting gait cycles, surgeons do have an additional tool to measure success after THA with regard on a healthy lifestyle. Maybe online measurements after discharge from hospital can be used to control and intervene if necessary to bring the patient back to an appropriate activity.

## Abbreviations

ASA: American Society of Anesthesiologists
BMI: Body mass index
GCs: Gait cycles
GEE: Generalized estimating equations
HHS: Harris Hip Score
MIS: Minimal invasive surgery
MVPA: Moderate-to-vigorous physical activity
PAL: Physical activity level
THA: Total hip arthroplasty
WOMAC: Western Ontario and McMaster Universities Arthritis Index
